# Integrated Molecular and Hematobiochemical Biomarkers for the Detection of Bovine Babesiosis in Holstein Calves

**DOI:** 10.3390/vetsci13020176

**Published:** 2026-02-10

**Authors:** Haifa Ali Alqhtani, Mohamed Marzok, Rasha Yassin Elkhidr, Ahmed A. Elsayed, Safaa M. Barghash, Ahmed L. El-Naggar, Mohamed T. Ragab, Ahmed I. Ateya, Fatmah Ahmed Safhi, Wafaa A. Osman

**Affiliations:** 1 Department of Biology, College of Science, Princess Nourah bint Abdulrahman University, P.O. Box 84428, Riyadh 11671, Saudi Arabia; haalqhtani@pnu.edu.sa (H.A.A.); faalsafhi@pnu.edu.sa (F.A.S.); 2 Department of Clinical Studies, College of Veterinary Medicine, King Faisal University, P.O. Box 400, Al Ahssaa 31982, Saudi Arabia; remola@kfu.edu.sa; 3 Animal and Poultry Nutrition Department, Desert Research Center, Mataria, Cairo 11753, Egypt; decernes@drc.gov.eg (A.A.E.); safaabarghash@drc.gov.eg (S.M.B.); ahmedmycol@yahoo.com (A.L.E.-N.); mohamedtaaat@drc.gov.eg (M.T.R.); latif.wafaa@yahoo.com (W.A.O.); 4 Department of Development of Animal Wealth, Faculty of Veterinary Medicine, Mansoura University, Mansoura 35516, Egypt

**Keywords:** *Babesia* spp., calves, diagnostic indicators, gene expression profiling, inflammatory response, PCR amplification

## Abstract

Bovine babesiosis is a widespread tick-borne parasitic disease that compromises calf health and productivity, causing substantial economic losses. This study evaluated the potential of molecular and hematobiochemical biomarkers for the detection of babesiosis in Holstein calves by analyzing the expression dynamics of selected immune and antioxidant genes and their association with blood-based indicators. Blood samples were collected from 243 fattening calves, classified as healthy (*n* = 180) or naturally infected (*n* = 63). Transcriptional levels of immune-related genes (*IL-2*, *IL-6*, *TNF-α*, and *MCP-1*) and antioxidant genes (*SOD3*, *CAT*, *GPX*, and *GST*) were measured alongside hematological, biochemical, immunological, and oxidative stress assessments. Infected calves exhibited significant upregulation (*p* < 0.05) of immune and pro-inflammatory genes, indicating strong immune activation, while antioxidant gene expression was markedly downregulated, reflecting impaired redox balance. These molecular changes were accompanied by hematobiochemical alterations, including elevated liver and kidney markers, serum lipids, inflammatory mediators, and oxidative stress indices. Conversely, reductions were observed in glucose, serum proteins, thyroid hormones, essential minerals, total antioxidant capacity, and endogenous antioxidant enzymes. The coordinated assessment of immune and antioxidant gene expression with hematobiochemical profiles provides a robust biomarker-based approach for the early detection and monitoring of bovine babesiosis. These integrated molecular and blood-based indicators may support effective diagnosis, disease management, and control strategies in livestock production systems.

## Simple Summary

Bovine babesiosis is a tick-borne parasitic disease that impairs the health and productivity of Holstein calves. This study investigated the link between immune- and antioxidant-related gene expression and blood and serum biochemical changes in naturally infected calves. Infected animals showed increased expression of immune genes and decreased expression of antioxidant genes, reflecting strong inflammatory responses and increased oxidative stress. These molecular changes coincided with hematological disturbances and altered biochemical markers of liver and kidney function, metabolism, mineral balance, and antioxidant status in the calves. The results highlight a close relationship between gene expression dynamics and blood-based indicators during *B. bovis* infection and suggest that combining immune and antioxidant genes with hematobiochemical biomarkers can serve as effective tools for disease detection, monitoring, and control in livestock.

## 1. Introduction

Bovine babesiosis is a worldwide disease caused by an intraerythrocytic protozoan, “*Babesia*”, belonging to the genus *Babesia* [1,2]. It primarily affects cattle but also infects a variety of domestic and wild animals, leading to increases in the morbidity and mortality rate in cattle [3,4]. *Babesia* is the second most prevalent parasite in mammalian bloodstreams after trypanosomes [5]. The OIE lists it as an emerging disease under the “B” category of diseases [6]. The presence of external parasites in the dairy industry results in financial losses due to their detrimental effects on animal health and productivity [7,8]. These effects include depriving cattle of necessary nutrients, decreasing feed intake and efficiency, increasing stress levels in animals, causing skin damage, and serving as carriers of various diseases [9]. Certain *Babesia* species are zoonotic and impact human health [10]. Human babesiosis occurs primarily through the bite of infected ixodid ticks, particularly *Ixodes* spp., although transmission via blood transfusion and, rarely, organ transplantation has also been reported [11]. The disease is globally distributed, with increasing incidence documented in North America, Europe, Asia, and parts of Africa, reflecting expanding tick habitats, climate change, and closer interactions between wildlife, livestock, and humans [12]. From a One Health perspective, domestic animals and wildlife serve as important reservoirs for zoonotic *Babesia* species, underscoring the need for integrated surveillance and control strategies that link animal health, vector ecology, and human health [13]. It is a significant disease in the Middle East since it can occasionally manifest acutely with significant clinical changes in animal productivity [14]. *Babesia bigemina*, *Babesia divergens*, and *Babesia bovis* are the most frequent species that infect cattle [15]. *B. bovis* and *B. bigemina* are the causative agents of the disease in Egypt [16,17]. The protozoon is spread from one animal to another (transovarial transmission) by cattle ticks. *Rhipicephalus microplus* and *Rhipicephalus annulatus* are responsible for the transmission of *B. bovis* and *B. bigemina* among cattle [6].

*Babesia* species induce acute disease mainly through two mechanisms: hemolysis and circulatory disruption [18]. The clinical manifestations of this disease include anorexia, weight loss, ruminal atony, dyspnea, depression, fever, anemia, hemoglobinuria, jaundice, diarrhea, abortion, and decreased milk production [19,20]. The side effects include unconsciousness, seizures, tremors, and muscle atrophy [21]. Neurological symptoms include circling, aggressiveness, nystagmus, hyperesthesia, convulsions and paralysis [18].

Hematobiochemical assessments offer valuable insights into an animal’s nutritional status, health, and physiological well-being. Therefore, these parameters serve as useful indicators for broadly evaluating an animal’s general health condition [22]. Alterations in blood parameters beyond the normal reference ranges can be detected, providing valuable information for differential diagnosis and offering insights into the severity of tissue damage and levels of infection [23].

Numerous physiological processes, including oocyte maturation, fertilization, embryo growth during pregnancy, parturition, and the start of premature labor, are associated with oxidative stress [24]. Cytokines play a major role in the induction and regulation of the immune inflammatory response [25]. By regulating cell growth, maturation, activation, proliferation, and differentiation, cytokines exert a substantial influence on the type, magnitude, and duration of immune responses [26].

Modern molecular genetic techniques are used as complementary tools for disease management and to enhance overall animal health [27]. Researchers have successfully identified genetic markers, particularly single-nucleotide polymorphisms (SNPs), associated with disease susceptibility and resistance in cattle [28,29,30]. This indicates the presence of variation in the adaptive capacity or resilience of the host genome in response to disease [31]. Antioxidants, SNPs, immune systems, and altered gene expression linked to bovine babesiosis are areas of ongoing research. The current study aimed to assess gene expression, SNPs, and hematobiochemical, immunological, and oxidative changes associated with bovine babesiosis.

## 2. Materials and Methods

### 2.1. Study Area and Livestock Management Conditions

**Figure 1:**
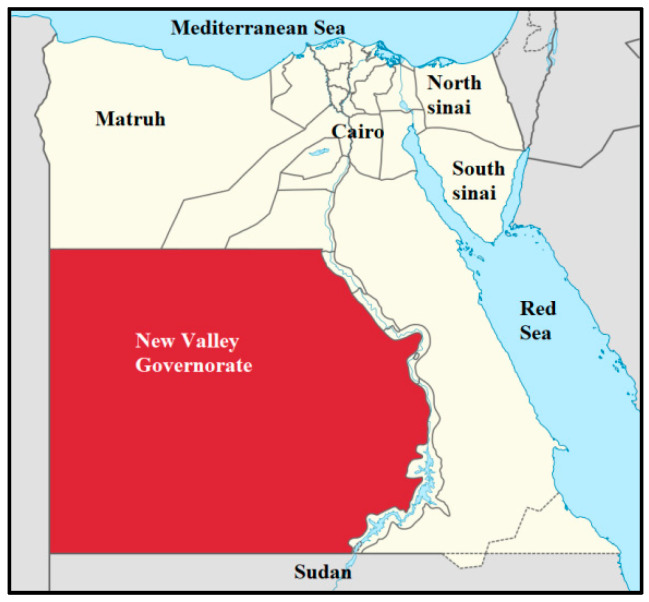
Geographical location of New Valley Governorate within Egypt.

The study was conducted in the New Valley Governorate, situated in Egypt’s western desert. This region forms a large depression bordered by the Nile, Sudan, and Libya (Figure 1). It spans approximately 440,098 km^2^ and lies between the coordinates 24°32′44″ N and 27°10′24″ E. The New Valley Governorate of Egypt, with Kharga Oasis as its administrative center, is characterized by a hyper-arid desert climate, high temperatures, and minimal annual rainfall, resulting in severe water scarcity and reliance on groundwater for livestock production. Livestock are predominantly managed under extensive grazing systems, with cattle, sheep, and goats freely grazing on desert pastures and oasis vegetation, while feed supplementation and veterinary services remain limited and irregular. These management practices, together with environmental stressors such as heat stress, nutritional deficiencies, and water depletion, increase livestock vulnerability to infectious diseases. The presence of irrigated agricultural areas, animal shelters, and traditional husbandry practices creates favorable microhabitats for ixodid ticks, particularly *Rhipicephalus* and *Hyalomma* species, which are well adapted to arid conditions. These ticks serve as the primary vectors of bovine babesiosis caused mainly by *Babesia bovis* and *Babesia bigemina*, which are enzootically maintained in local cattle populations. Epidemiological evidence from the region indicates high tick infestation rates and increased Babesia transmission during warmer months, highlighting the endemic nature of bovine babesiosis and the importance of understanding local tick ecology for effective disease surveillance and control.

### 2.2. Animals and Study Design

The current study comprised 243 fattening calves between December 2023 and May 2024, with an average age of 3 ± 0.3 years (1.5–5 years) and an average body weight of 317 ± 18.3 kg (215–400 kg). Calves were categorized based on their health status. The control group (G1) comprised 180 clinically healthy animals. The inclusion criteria for this group were no previous or current tick exposure, absence of any clinical abnormalities, and negative Babesia results confirmed by blood smear examination and PCR testing. Group 2 comprised 63 calves diagnosed with babesiosis. The enrollment criteria included observable tick infestation, the presence of clinical signs such as anorexia, weight loss, ruminal atony, dyspnea, depression, fever, anemia, pale or icteric mucous membranes, hemoglobinuria, and jaundice, along with laboratory confirmation of intra-erythrocytic Babesia stages via Giemsa-stained blood smears and Babesia-specific polymerase chain reaction (PCR). Positive samples with PCR were introduced to confirm the dominant strain of Babesia subspecies present in the study area through gene sequencing and phylogenetic analyses and recorded in the GeneBank database.

The calves were fed a basic diet designed for beef cattle and kept in partially shaded quarters [32]. The initial diet comprised 40% wheat straw and 60% concentrate. Free water was provided along with a diet twice a day, in the morning and evening. In accordance with the Egyptian Authority Program, the animals were routinely vaccinated and dewormed. A thorough clinical examination was performed on each calf, and clinical symptoms were noted. All cases included in this study were free from bacterial, viral, helminth, and protozoan parasites, as animals were subjected to clinical examination to eliminate bacterial and viral infected cases as much as possible [33], and all suspicious cases were denied from the study. Fecal samples were obtained directly from the rectum, packed in clean plastic containers, numbered, and transferred to the laboratory for parasitological examination to eliminate cases with helminth or protozoal parasites. The Ministry of Agriculture and Land Reclamation of Egypt and the Animal and Poultry Health Department at the Desert Research Center (IACUC-APPD-DRC-# 25 8 51 #) instructed that the study adhere to all relevant national regulations and institutional guidelines for the use and care of animals. The protocols followed the All experimental procedures were conducted in accordance with the ARRIVE guidelines and the International Animal Ethics Committee standards (8th Edition, 2011; Record No.: 13799; Legacy ID: 8247) and were approved by the institutional animal care and use committee.

### 2.3. Blood Sampling

First, 10 mL of blood was collected from each calf using jugular venipuncture. The samples were divided into plain tubes without anticoagulant and EDTA-containing tubes for serum and whole-blood analyses, respectively. All samples were placed on crushed ice and immediately transported to the laboratory for processing. EDTA-treated whole blood was used for complete blood count (CBC) analysis and for RNA extraction. Blood collected in plain tubes was allowed to clot overnight at room temperature and centrifuged at 3000 rpm for 15 min. The separated serum was aliquoted, stored at −20 °C, and subsequently used for biochemical assays of oxidative and energy stress biomarkers.

### 2.4. Parasitological Examination

From each blood sample, two thin smears were made, allowed to air-dry, and fixed in absolute methanol for 5 min. The slides were then stained with 10% Giemsa for one hour. Microscopic examination was performed using a light microscope with an oil-immersion objective (×1000), following the procedure described in [34]. Identification of the parasites was based on the morphological features detailed in [35].

### 2.5. PCR Identification and Genotyping of Babesia

Genomic DNA was isolated from whole blood using the DNeasy Blood and Tissue Kit (Qiagen, Hilden, Germany) following the manufacturer’s protocol. Only samples that tested positive or negative by blood smear evaluation were further analyzed using a PCR assay to verify the presence of Babesia. Amplification targeted the 18S rRNA gene using species-specific primers: forward (GTCTTGTAATTGGAATGATGGTGAC) and reverse (ATGCCCCCAACCGTTCCTATTA), which produced a 340 bp fragment as described by [36]. PCR amplification was performed in a final reaction volume of 25 µL containing 12.5 µL of commercial Master Mix (Bio-Basic, Alverca do Ribatejo, Portugal), 10 pmol of each primer, approximately 25 ng of genomic DNA, and sterile distilled water. Reactions were carried out using an automated thermocycler (Bio-Rad, Hercules, CA, USA). The thermal profile consisted of an initial denaturation at 94 °C for 5 min, followed by 30–40 cycles of denaturation at 94 °C for 30 s, annealing at 55 °C for 40 s, and extension at 72 °C for 40 s, with a final extension step at 72 °C for 10 min.

PCR products were resolved by agarose gel electrophoresis and visualized using a gel documentation system (Alpha Innotech, San Leandro, CA, USA). The presence of a 340 bp band was considered indicative of *Babesia* species infection. Purified amplicons were subsequently sequenced using an Applied Biosystems 3130 automated DNA sequencer (ABI, Los Angeles, CA, USA). Sequencing reactions were prepared with BigDye Terminator v3.1 (2 µL), primer (1 µL), and variable volumes of template DNA (1–10 µL) depending on the band quality and DNA concentration, with the final reaction volume adjusted to 20 µL using deionized or PCR-grade water. The obtained sequences were analyzed using BLAST (BLASTn version 2.12.0+ (National Center for Biotechnology Information, NCBI, Bethesda, MD, USA) to determine sequence identity and were compared with reference sequences deposited in GenBank [37] and then compared with those of closely related species preserved in the GenBank database. A phylogenetic tree was created using the neighbor-joining model in MEGA Version 6.0 software [38].

### 2.6. RNA Extraction, cDNA Synthesis, qRT-PCR, and Polymorphism Detection

Total RNA was isolated from EDTA-treated blood samples using Trizol reagent (RNeasy Mini Kit, Qiagen, Hilden, Germany). The RNA concentration and quality were assessed with a NanoDrop spectrophotometer Quawell, CA, USA. cDNA synthesis was performed using the RevertAid First Strand cDNA Synthesis Kit (Thermo Fisher, Cat. No. EP0441, London, UK). Quantitative real-time PCR (qRT-PCR) was carried out with SYBR Green Master Mix (SensiFAST, Bioline, SensiFast™ SYBR, Bioline, Cat. No. Bio-98002, London, UK) to evaluate the expression of immune-related genes (*IL-2*, *IL-6*, *TNF-α*, and *MCP-1*) and antioxidant genes (*SOD3*, *CAT*, *GPX*, and *GST*), with *GAPDH* used as the internal control. Primer details are provided in Table 1, and relative gene expression was calculated using the 2^–ΔΔCt^ method [39].

PCR amplicons were purified using a commercial purification kit (Jena Bioscience, Jena, Germany), quantified using a NanoDrop Q5000 spectrophotometer (Thermo Fisher, Waltham, MA, USA), and subsequently sequenced on an ABI 3730XL DNA Analyzer (Applied Biosystems, Waltham, MA, USA). Forward and reverse reads were assembled and aligned using Chromas 1.45, and sequence identity was confirmed using BLAST analysis [37]. Single-nucleotide polymorphisms (SNPs) were detected by comparing the obtained sequences with the corresponding GenBank reference entries. Amino acid alignments and comparative analyses were performed using MEGA6 software version 6 [40].

### 2.7. Biochemical Analysis

Serum biochemical parameters were measured using commercial diagnostic kits following the manufacturers’ standard protocols. Total protein, albumin, glucose, cholesterol, and creatinine concentrations were determined using kits from Gamma Trade Company (Mohandessin, Giza, Egypt), while calcium levels were assessed using Bio-Diagnostic reagents (Giza, Egypt). Sodium, potassium, triglycerides, urea, AST, ALT, LDH, and GGT were quantified using Spectrum kits, (Obour City, Cairo Governorate, Egypt) on an automated chemistry analyzer (Apple 302, Cupertino, CA, USA). Total bilirubin was evaluated using N.S. BIO-TEC kits, (Camp Shizar, Alexandria Governorate, Egypt), and cardiac troponin I (cTnI) was measured using a MyBioSource kit (MyBioSource, San Diego, CA, USA). Globulin concentrations were calculated by subtracting albumin from total protein. Trace minerals were analyzed using the following kits: copper (SIGMA-ALDRICH, St. Louis, MI, USA), zinc (Abnova, Neihu District, Taipei, Taiwan), and iron (Abcam, Cambridge, UK). Oxidative and antioxidant markers—including malondialdehyde (MDA; Biodiagnostic, CAT No: MD2529), nitric oxide (NO; CAT No: NO2533), superoxide dismutase (SOD; CAT No: SD2520), total antioxidant capacity (TAC; CAT No: TA2513), reduced glutathione (GSH; CAT No: GR2511), and catalase (CAT; Biodiagnostic, Dokki, Giza Governorate, Egypt)—were also quantified. Cytokines were measured using ELISA kits as follows: IL-2 (RayBiotech, Catalog #: ELB-IL2-1), IL-6 and IL-10 (Boster Biological Technology, Pleasanton, CA, USA. CAT No: EK0412 and EK0418, respectively), and TNF-α and IFN-γ (Aviva Systems Biology, San Diego, CA, USA). Serum amyloid A (SAA) was assayed using IBL International (Toronto, ON, Canada) ELISA kits, while C-reactive protein (CRP) was determined using a RayBiotech kit (Catalog #: ABIN6954219). MCP-1 (Monocyte chemoattractant protein-1) and T4 ELISA Kit (MyBioSource, San Diego, CA, USA, Catalog #: MBS454447, MBS8819985), respectively.

### 2.8. Statistical Analysis

Statistical analyses were performed using SPSS version 23. Independent-samples t-tests were applied to compare the mean values of measured variables between infected and control groups, with results expressed as mean ± SD. The chi-square test was used to examine differences in the frequencies of each gene SNP between infected and healthy calves, evaluating the distribution of identified SNPs across the groups. The observed genotype frequencies were subjected to a chi-square (χ^2^) test to assess Hardy–Weinberg equilibrium and to characterize genotype distribution in the study population [41,42]. Furthermore, Linear Discriminant Analysis (LDA) was conducted to assess whether the average SNP values of the genes could distinguish between infected and healthy animals. The mean scores of the eight genes served as predictor variables, while health status (infected vs. healthy) was used as the grouping variable. A significance level of *p* = 0.05 was applied for all tests.

## 3. Results

### 3.1. Clinical Examination

Clinical evaluation of the 243 examined calves showed that 180 animals (74.07%) were clinically healthy. These calves displayed normal physiological parameters, including body temperature, pulse, and respiratory rate, and exhibited bright, clear eyes without discharge, a moist muzzle, and no evidence of internal or external parasitism. They demonstrated normal posture, appetite, and behavior, with no signs of diarrhea, lameness, or abnormal lung sounds upon auscultation. Additionally, they had no current or prior history of tick infestation and showed no clinical indicators suggestive of babesiosis. Conversely, 63 calves (25.93%) presented with clinical abnormalities. These included anorexia, weight loss, ruminal atony, dyspnea, depression, fever (≥39.5 °C), anemia, pale or icteric mucous membranes, hemoglobinuria, and jaundice, along with a documented history of previous tick exposure. Microscopic examination of Giemsa-stained blood smears confirmed the presence of Babesia, characterized by the typical intraerythrocytic, double pear-shaped (pyriform) forms. Analysis of the study population revealed that there was no significant association between the age of the animals and their body condition scores (*p* > 0.05). Both younger and older animals displayed a wide range of body conditions, indicating that age was not a determining factor for nutritional status in this population. These findings suggest that other factors, such as feeding practices, environmental stressors, and management conditions, are likely more influential in affecting the body condition of livestock in the New Valley Governorate.

### 3.2. Detection and Genotyping of Babesia

**Figure 2:**
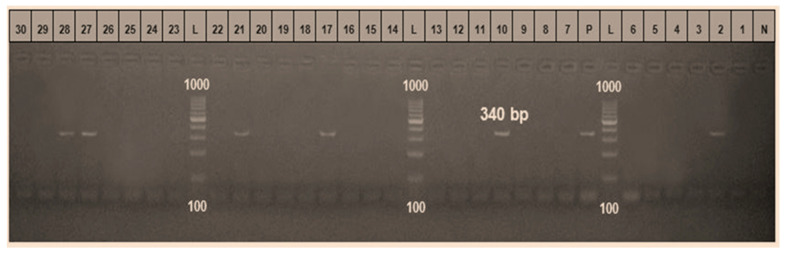
Representative 1.5% agarose gel electrophoresis showing positive amplification of the 18S rRNA gene of *Babesia* spp. Positive samples are shown in lanes 2, 10, 17, 21, 27, and 28. Lane P represents the positive control, lane N the negative control, and lanes L contain the molecular size marker (Fermentas, Darmstadt, Germany). All remaining lanes show negative results.

The PCR-based assay confirmed the results of clinical and blood film examinations. However, DNA amplified for *Babesia* species was detected in 63 calves, producing bands with sizes of 340 bp, while 180 had no infections, as shown in Figure 2 (original image is shown in Figure S1). The results also showed that the fragments generated by PCR had the same sequence as *B. bigemina* with a typical identity of 100% after gene sequencing analysis. No genetic diversity, different SNPs, divergence, or genetic distances between them have been determined so far. The submitted strain had the same sequence as other preserved *B. bigemina* strains in GenBank. The sequence was recorded under accession number PP892245 *B. bigemina* BFNV-DRC in the GenBank database.

### 3.3. Hematological Profile

When comparing calves infected with *B. bigemina* to healthy control animals, a substantial (*p* < 0.05) decrease in the mean RBCs, Hb, PCV%, MCHC, WBCs, lymphocyte, and neutrophil counts was noted, along with an increase in MCV (Table 2).

### 3.4. Patterns for Transcript Levels of Immune and Antioxidant Indicators

**Figure 3:**
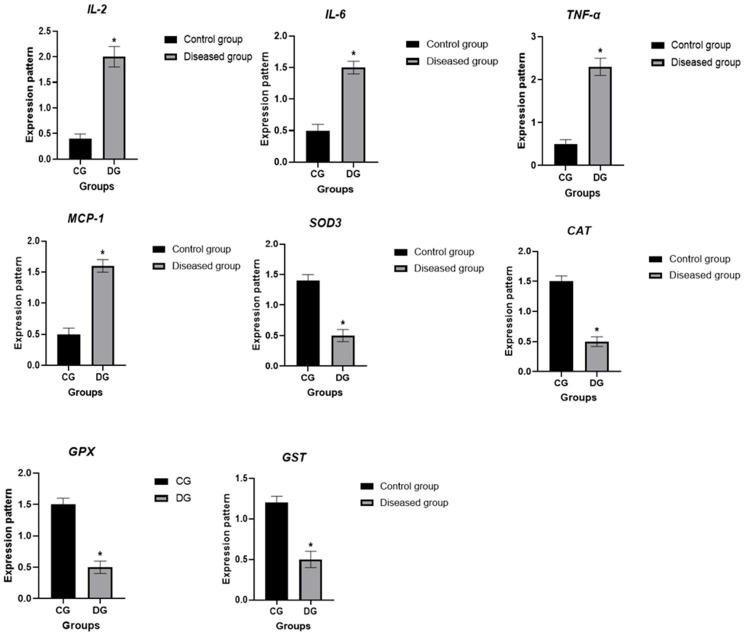
Differential expression of immune-related genes (*IL-2*, *IL-6*, *TNF-α*, and *MCP-1*) and antioxidant genes (*SOD3*, *CAT*, *GPX*, and *GST*) in *Babesia*-affected calves. Data are presented as mean ± SE. An asterisk (*) indicates statistically significant differences (*p* < 0.05).

Figure 3 illustrates the transcript patterns of the evaluated immune and antioxidant markers. Calves infected with *Babesia* exhibited significantly higher expression levels of *IL-2*, *IL-6*, *TNF-α*, and *MCP-1* than healthy calves. In contrast, the expression of the antioxidant genes *SOD3*, *CAT*, *GPX*, and *GST* was notably lower in the infected group. Among the infected calves, TNF-α showed the highest mRNA expression (2.34 ± 0.24), whereas *CAT* showed the lowest (0.51 ± 0.08). In healthy calves, *GPX* demonstrated the highest mRNA level (1.58 ± 0.12), whereas *IL-2* had the lowest (0.45 ± 0.09).

### 3.5. Genetic Polymorphisms of Immune and Antioxidant Genes

The PCR-amplified DNA fragments for *IL-2* (480 bp), *IL-6* (400 bp), *TNF-α* (359 bp), *MCP-1* (399 bp), *SOD3* (343 bp), *CAT* (423 bp), *GPX* (461 bp), and *GST* (419 bp) showed sequence variations between healthy calves and *Babesia*-infected calves. Comparison with reference sequences from GenBank confirmed all detected SNPs in the immune and antioxidant genes.

The exonic changes identified in these genes, summarized in Table 3, resulted in coding sequence alterations in infected calves compared to those in healthy calves. Sequencing analysis identified 14 SNPs, including eight synonymous and six non-synonymous variants. Significant differences in SNP frequencies were observed between the infected and healthy calves (*p* < 0.005). Chi-square tests demonstrated that the distribution of these SNPs across all genes differed significantly between the two groups, with the overall chi-square value indicating substantial variation between resistant and infected animals (*p* < 0.05) (Table 3).

The Hardy–Weinberg equilibrium (HWE) was assessed for each SNP locus independently in both the healthy control and *Babesia*-infected groups. The chi-square (χ^2^) analysis demonstrated that genotype frequencies at all examined loci conformed to genetic equilibrium in both populations, as the calculated χ^2^ values were lower than the corresponding tabulated χ^2^ values (Table 4).

Table 5 presents the discriminant analysis results, illustrating the association between gene variants and calves’ health status. The model successfully classified all calves as either healthy or infected, demonstrating that the SNP markers included have strong discriminatory power and may be valuable as potential genetic indicators of susceptibility to *B. bovis* infection.

### 3.6. Biochemical Profile

The biochemical results showed that serum levels of cholesterol, triglycerides, urea, creatinine, total bilirubin, K, and serum activity of AST, ALT, GGT, and LDH were considerably higher (*p* < 0.05) in diseased calves than in non-infected ones, while serum levels of glucose, total protein albumin, globulin, T4, cTnI, calcium, sodium, copper, zinc, and iron were significantly lower (Table 6).

### 3.7. Antioxidants and Immunological Profile

When comparing infected calves to healthy calves, the oxidative stress biomarkers MDA (1.50 ± 0.008 mg/dL) and NO (6.4 ± 0.1 µmol/L) exhibited a significant (*p* < 0.05) increase, while TAC (62.4 ± 0.6 mg/dL), GSH (16.7 ± 0.7 mg/dL), SOD (25 ± 0.5 U/mL), and CAT (25 ± 0.5 U/mL) revealed a significant (*p* < 0.05) decrease (Table 6). Serum concentrations of TNF-α (150.7 ± 2.6 pg/mL), IFN-γ (118 ± 2 pg/mL), IL2 (167.6 ± 4.7 pg/mL), IL6 (90 ± 38 pg/mL), IL10 (119 ± 1.1 pg/mL), MCP-1 (125 ± 0.5 pg/mL), SAA (8 ± 0.08 mg/L), and CRP (9.6 ± 0.4 g/L) were significantly higher (*p* < 0.05) in infected calves (PG) than in the healthy group (Table 7).

## 4. Discussion

Bovine babesiosis is a major endemic disease in Egypt, where its acute form severely reduces productivity and causes substantial economic loss [43,44]. Globally, approximately 1.2 billion cattle in regions, including Asia, Australia, Africa, South and Central America, and the United States, are at risk [45]. Developing effective vaccines against *Babesia* spp. remains challenging because of the limited understanding of the pathophysiology of the disease and the lack of well-defined immunological correlates of protection.

### 4.1. Clinical Findings

The sudden onset of pyrexia observed in this study may result from the effects of toxic metabolites produced during *Babesia* metabolism on thermoregulation [33]. Additionally, hemolysis of red blood cells leads to anemia and anemic hypoxia, triggering increased heart and respiratory rates as a compensatory response to tissue oxygen deficiency [33]. Severe hemolysis can also cause hemoglobinemia and hemoglobinuria in infected animals [46]. Overall, the clinical signs observed here align with previous reports of babesiosis [47,48,49].

### 4.2. Parasitological Findings

The present study found a *Babesia* infection rate of 25.93% in the study area, which varies compared with previous reports in Egypt using different detection methods. Infection rates were 18.43% in camels on the northwest coast [50] and 2.81% in southeastern Egypt [51], 51.28% in cattle and 22.47% in buffaloes in El-Qalubia [51], and 10% in Beheira and Faiyum Governorates [52]. In the middle Delta, prevalence was 12.6%, compared with lower rates in the west Delta and Upper Egypt (5.3% and 3.97%) [52]. Internationally, *B. bigemina* was detected in 92.6% of calves and 84% of cows in Brazil [53], 11.1% in Tunisia [54], and nested PCR studies reported 18.8% in Pakistan [55], 78.5% in Portugal [56], 29% in Brazil [57], and 26.7% in the Philippines [58].

In the present study, *B. bigemina* was the predominant *Babesia* species in calves, whereas previous studies in Egypt reported *B. bovis* as dominant in the northwest coast [50], southeast Egypt [51], and South Sinai [59]. The prevalence of *B. bigemina* in our study may reflect local regulations restricting animal imports from other governorates to prevent disease introduction. The strain identified was 100% identical to other *B. bigemina* sequences in GenBank, including EF458190 from buffaloes in South Africa [60], HQ264114 from white-tailed deer in the USA [61], JQ437261 from cattle, and EF458194 from buffaloes in Australia [62], as well as MN227676 previously isolated from cattle in Assiut, Egypt [44], indicating a widespread global distribution.

### 4.3. Hematological Profile

In this study, *Babesia bigemina*-infected calves exhibited macrocytic hypochromic anemia, evidenced by significant reductions in mean RBC count, hematocrit (HCT%), hemoglobin concentration, and MCHC, along with an increase in MCV. These findings are consistent with previous reports [20,49,63]. The anemia may result from multiple pathogenic mechanisms, including the production of anti-erythrocyte antibodies that target both infected and uninfected red blood cells [64], mechanical damage caused by binary fission of intra-erythrocytic trophozoites, and increased erythrophagocytosis by activated macrophages [65]. Furthermore, the observed macrocytic hypochromic pattern likely reflects a compensatory response to hemolysis, characterized by the extensive release of immature reticulocytes from the bone marrow and accelerated erythropoiesis [66].

In the present study, *Babesia*-infected calves exhibited leukopenia, as reflected in the leucogram. This reduction in white blood cell counts may be partially explained by the interaction between platelets and activated endothelial cells, which promotes “secondary capture” of leukocytes and contributes to their decreased circulation [67]. Neutropenia, in particular, may result from multiple mechanisms, including splenic sequestration, increased adherence of neutrophils to the endothelium, direct damage to hematopoietic precursor cells, or a combination of these factors [68]. Such neutropenia is commonly observed during acute infections [69]. Lymphopenia in infected calves may also be associated with stress-induced immunosuppression [70], while overall leukopenia may further reflect the sequestration of both neutrophils and lymphocytes in the spleen [71]. Collectively, these findings highlight the multifactorial nature of leukocyte depletion during acute *Babesia* infection.

### 4.4. Genetic Polymorphisms Between Healthy and Babesiosia Affected Cows

The idea implies that chromosomal polymorphisms affect the expression levels of mRNA for their respective genes, serving as a heritable endophenotype [72]. This approach also backed up the notion that integrating information on gene expression and chromosomal abnormalities could improve our comprehension of the genetics underpinning disease onset [73].

The mRNA expression profiles of immune-related genes (*IL-2*, *IL-6*, *TNF-α*, and *MCP-1*) and antioxidant genes (*SOD3*, *CAT*, *GPX*, and *GST*) were evaluated to assess differences in immune and antioxidant status between healthy calves and those infected with *Babesia*. Infected calves exhibited markedly higher expression of the immune genes, while the antioxidant genes were expressed at lower levels compared to healthy calves.

These findings are consistent with previous studies showing that *Babesia* infection in calves induces early upregulation of type 1 cytokines such as *IL-12* and *IFN γ* [74,75]. The observed SNP differences may also influence host susceptibility, supporting prior reports that host genetic variation shapes immune responses during babesiosis [76]. Our observations of increased *IL-2*, *IL-6*, *TNF-α*, and *MCP-1* expression align with previous findings in bovine babesiosis [74,76] and canine models [77], suggesting that these cytokines are central mediators of leukocyte recruitment and parasite clearance. The oxidative stress induced by ROS and NO may further amplify tissue inflammation, as reported in calves and small ruminants during *Babesia* infection [16,77]. The downregulation of *SOD3*, *CAT*, *GPX*, and *GST* in infected calves mirrors earlier studies demonstrating reduced antioxidant enzyme activity during babesiosis in calves and sheep [16]. This suggests that oxidative stress contributes to disease pathophysiology and may influence clinical severity.

In this study, we investigated the immune-related genes (*IL-2*, *IL-6*, *TNF-α*, and *MCP-1*) and antioxidant genes (*SOD3*, *CAT*, *GPX*, and *GST*) in calves both infected and uninfected with *Babesia* using PCR-based DNA sequencing. The analysis revealed distinct SNP profiles between the two groups. Compared to reference sequences from GenBank, the polymorphisms identified here provide novel insights into the genetic variation in these markers. The role of SNPs in immune and antioxidant genes in influencing susceptibility to babesiosis in calves has not been previously explored. Our study, using Bos taurus gene sequences published in PubMed, is the first to demonstrate a link between these genetic variations and the host response to babesiosis.

All analyzed SNP loci in both Babesia-infected and healthy calves conformed to Hardy–Weinberg equilibrium, indicating genetic stability of the studied populations and reliability of genotype data. This equilibrium supports the validity of the observed polymorphisms and suggests that the identified variations in immune and antioxidant genes reflect true biological diversity rather than sampling or technical bias, reinforcing their potential role in host response to babesiosis [42,78].

Importantly, this research represents the first application of combined gene expression profiling and RT-PCR DNA sequencing of immune and antioxidant genes to assess host genetic responses in dairy calves, whereas similar candidate gene strategies have previously been employed to study trypanosomiasis in dromedary camels [79]. In camels infected with trypanosomiasis, significant upregulation was observed in the genes *TLR2*, *TLR5*, *IL-17*, *MARCHF3*, *RASGRP1*, *EPS15L1*, *PPIE*, *ASB16*, *CMPK2*, *LPCAT1*, *FPGT*, *GPHN*, *TNNI3K*, *DIO3*, *Keap1*, and *OXSR1*, while the genes *Nrf2*, *PRDX6*, and *NDUFS5* were notably downregulated. PCR-based DNA sequencing was conducted to identify nucleotide polymorphisms across immune genes (*TLR2*, *TLR5*, *IL-17*, *MARCHF3*, *RASGRP1*, *EPS15L1*), metabolic genes (*PPIE*, *ASB16*, *CMPK2*, *LPCAT1*, *FPGT*, *GPHN*, *TNNI3K*, *DIO3*), and antioxidant genes (*Nrf2*, *Keap1*, *PRDX6*, *NDUFS5*, *OXSR1*) in order to compare genetic variations between healthy camels and those affected by trypanosomiasis.

### 4.5. Biochemical Profile

In our study, Babesia-infected calves exhibited significantly lower serum glucose levels compared to healthy controls. This hypoglycemia may result from multiple factors, including increased glucose utilization by the parasites, hepatic dysfunction associated with Babesia infection, and reduced feed intake due to anorexia and refusal to graze [80]. These findings are consistent with previous reports documenting decreased blood glucose in infected cattle [81]. Conversely, serum triglycerides and cholesterol levels were significantly elevated in infected calves. Similar observations have been reported in other studies [82], and the increase in triglycerides may reflect enhanced hepatic synthesis or impaired clearance from the circulation. Furthermore, elevated triglyceride levels during the acute phase of infection may represent a component of the host’s metabolic and immune response to Babesia [83].

In the present study, serum levels of total protein, albumin, and globulin were significantly reduced in Babesia-infected calves. This decline may result from impaired liver function caused by the parasite’s direct and indirect effects, as well as from digestive disturbances such as diarrhea, reduced feed intake, and fever [16]. Specifically, decreased albumin levels may arise from reduced hepatic synthesis, leakage from damaged red blood cells, or urinary losses [84]. These findings are in agreement with previous studies reporting hypoproteinemia and hypoalbuminemia in Babesia-infected cattle [20]. Additionally, research indicates that crossbred cattle under improved management practices may have less prior exposure to vectors and limited immunity, rendering them more susceptible to clinical infection when exposed [85].

In our study, Babesia-infected calves exhibited significant elevations in liver and renal enzymes, including AST, ALT, GGT, LDH, urea, and creatinine, indicating hepatic and renal dysfunction. The increase in AST and ALT levels may result from damage to hepatocytes, skeletal muscle, or red blood cells caused by the toxic metabolites of *Babesia* sp. [86]. Similarly, elevated BUN and creatinine levels likely reflect renal impairment, potentially due to glomerulonephritis induced by the parasitic infection [87]. The observed rise in total bilirubin in infected calves may be attributed to a combination of hemolytic crisis and hepatic injury [88]. Overall, these findings corroborate previous reports demonstrating liver and kidney involvement in babesiosis, confirming the systemic impact of the infection on affected calves [48,81,89,90,91].

In the present study, Babesia-infected calves exhibited a significant decrease in serum thyroxine (T4) levels. This reduction may be attributed to increased metabolic demand, as T4 is converted to the more metabolically active triiodothyronine (T3), resulting in lower circulating T4 concentrations [92]. These findings align with previous reports, which also documented decreased T4 levels in calves under similar infectious or metabolic stress conditions [93].

In this study, Babesia-infected calves exhibited significantly higher serum potassium levels and lower concentrations of calcium, sodium, zinc, copper, and iron compared to healthy controls. These alterations are consistent with previous reports [49,81,94] and may be attributed, in part, to degeneration and necrosis of renal convoluted tubules caused by the parasite [16]. The reduction in calcium levels could also result from decreased dietary intake or disruption of thyroid function induced by irritation from external parasites [95]. Cu and Zn help the body’s antioxidant system by avoiding damage from radicals [96]. While the precise mechanism by which zinc protects against redox-active substances remains unclear, copper has been demonstrated to play a protective role in bovine babesiosis [43]. For instance, Cu–Zn superoxide dismutase (SOD) catalyzes the conversion of O_2_ to H_2_O_2_, acting as both an antioxidant and pro-oxidant to reduce free radical-induced cellular damage [97,98,99]. These findings highlight the interplay between mineral imbalances and oxidative stress in the pathophysiology of Babesia infection.

### 4.6. Antioxidant Profile

Compared with healthy animals, infected calves exhibited significantly elevated levels of oxidative stress markers (NO and MDA) along with significantly reduced antioxidant parameters (GSH, SOD, CAT, and TAC). These results are consistent with previous studies [77,91].

Parasitic infections activate inflammatory cells that play a crucial role in host defense. This activation induces pro-inflammatory cytokines, including tumor necrosis factor-α (TNF-α), interleukin-1β (IL-1β), interleukin-6 (IL-6), interleukin-12 (IL-12), and interferon-γ (IFN-γ), which regulate oxidant-generating enzymes [100,101]. In bovine babesiosis caused by *Babesia bigemina*, infected erythrocytes stimulate macrophage activation, leading to increased inducible nitric oxide synthase (iNOS) expression and nitric oxide production. The resulting release of reactive nitrogen intermediates enhances oxidative stress, promotes lipid peroxidation, and increases malondialdehyde (MDA) levels [101]. Elevated inflammatory cytokine production promotes the generation of reactive oxygen and nitrogen species, including hydrogen peroxide, nitric oxide, superoxide anion, and hydroxyl radicals, which can damage proteins, lipids, carbohydrates, and DNA [100]. In the present study, MDA and NO levels were significantly higher in diseased cattle, consistent with findings in *Anaplasma marginale*-infected calves [102]. This increase is closely associated with infection severity, oxidative stress, and depletion of antioxidant defenses, further supporting previous reports of elevated MDA and NO levels in bovine anaplasmosis [103]. Infected calves showed significantly decreased levels of GSH, CAT, SOD, and TAC. The reduction in GSH may be attributed to its increased utilization in neutralizing reactive oxygen species or enhanced glutathione peroxidase activity during *Anaplasma marginale* infection [104]. Similarly, the decline in TAC likely reflects the depletion of antioxidant defenses under infection-induced oxidative stress, as these enzymes function as key free radical scavengers [101].

### 4.7. Immunological Profile

In the present study, babesiosis induced cytokine production to initiate an immune response. The presence and proliferation of *Babesia bigemina* stimulated immune cells to release pro-inflammatory cytokines in response to tissue damage [105]. Compared with controls, infected calves exhibited significantly elevated serum levels of TNF-α, IFN-γ, MCP-1, SAA, CRP, IL-2, IL-6, and IL-10, indicating activation of both innate and adaptive immune responses.

In the *Babesia*-infected group, serum CRP and SAA levels were significantly elevated, suggesting an IL-6-mediated inflammatory response in babesiosis. Consistent with this, low circulating CRP has been proposed as an in vivo marker of inhibited IL-6 activity due to increased free anti-IL-6 receptor antibodies [106]. IL-10, a key regulatory cytokine, was significantly elevated in *Babesia*-infected calves. Elevated IL-10 levels have been associated with excessive inflammatory responses and poor prognosis, likely due to infection-induced immunosuppression, particularly when the IL-10/TNF-α ratio is increased [107,108].

MCP-1 levels were significantly elevated in Babesia-infected calves, with higher concentrations associated with poor prognosis, highlighting its role in regulating the host immune response [109]. MCP-1 has shown the strongest association with organ dysfunction and mortality in sepsis compared with other cytokines and is markedly elevated in critically ill dogs, immune-mediated hemolytic anemia, myxomatous mitral valve disease, and various cancers, where it also serves as a prognostic marker [110,111,112,113]. Consistent with previous studies [79,114], our findings support the prognostic significance of MCP-1.

## 5. Conclusions

According to the results shown here, bovine babesiosis is associated with notable immunological and antioxidant alterations, particularly in blood levels of TNF-α, IFN-γ, IL2, IL6, IL10, MCP-1, SAA, CRP, MDA, NO, TAC, GSH, SOD, and CAT. Our findings highlight that variations in nucleotide sequences and gene expression patterns of the studied immune and antioxidant genes play a crucial role as risk factors and are closely associated with calves’ susceptibility to Babesia infection. These results indicate that there is genetic diversity among animals and present new genetic markers and prospective candidate genes for detecting Babesia infection in calves. These indicators offer potential opportunities to prevent the disease through selective breeding programs and could be utilized as reliable stand-ins for babesiosis in calves.

## Figures and Tables

**Table 1:** Sequences of forward and reverse primers used for real-time PCR analysis of the immune- and antioxidant-related genes investigated in this study.

Investigated Marker	Primer	Product Size (bp)	Annealing Temperature (°C)	GenBank Isolate
*CAT*	F: 5′-TCTGTTGAAGATGCAGCAAGAC-3′ R: 5′-GCCGTCACGCTGGTAGTTGGCCA-3′	435	60	NM_001035386.2
*GAPDH*	F: 5′-AGGTCGGAGTGAACGGATTC-3′ R: 5′-GATGTTGGCAGGATCTCGCT-3′	242	58	NM_001034034.2
*GPX*	F: 5′-GAGAACGTAGCATCGCTCTGAG-3′ R: 5′-CACACCGTCTGGGCCCACCAGGA-3′	396	58	NM_174076.3
*GST*	F: 5′-TCCAATGCCATCCTGCGGCACCT-3′ R: 5′-CTGTTTCCCATTGCCGTTGATGG- 3′	435	58	NM_177516.1
*IL-2*	F: 5′-TACAAGATACAACTCTTGTCT-3′ R: 5′-TCATATTCACATGTGAATCTTGT-3′	386	58	NM_180997.2
*IL-6*	F: 5′-ACAAGCGCCTTCACTCCATTCG-3 R: 5′-AGACTGCATCTTCTCCAGCAGG-3′	516	60	EU276071.1
*MCP-1*	F: 5′-ATGAAGGTCTCCGCTGCCCTCCT-3′ R: 5′-AGGCTTTGGAGTTTGGTTTTTC-3′	297	60	NM_174006.2
*SOD3*	F: 5′-GTGCTGCCGTCGGCCACGCTG-3′ R: 5′-GACGGCTTTACACTCGCTCTCGC-3′	528	58	NM_001082610.2
*TNFα*	F: 5′-AGCATGATCCGGGATGTGGAGC-3′ R: 5′-GACTCCTCCCTGGTAGATGGGT-3′	588	60	AF348421.1

Catalase (*CAT*), Glyceraldehyde-3-phosphate dehydrogenase (*GAPDH*); Glutathione Peroxidase (*GPX*), Glutathione S-Transferase (*GST*), Interleukin-2 (*IL-2*), Interleukin-6 (*IL-6*), Monocyte Chemoattractant Protein-1 (*MCP-1*), Superoxide Dismutase 3 (*SOD3*), and Tumor Necrosis Factor-alpha (*TNF-α*). A: Adenine, T: Thymine, C: Cytosine, G: Guanine.

**Table 2:** Comparison of hematological parameters between apparently healthy (*n* = 180) and *Babesia*-infected (*n* = 63) calves in the New Valley Governorate, Egypt.

Parameters	Healthy Calves	Infected Calves	*p* Values	Unit
RBCs	6.3 ± 0.6	4.2 ± 0.5 *	0.001	×10^6^/µL
Hb	11.9 ± 1.5	8.1 ± 1.01 *	0.001	g/dL
PCV	32.1 ± 0.5	22 ± 0.3 *	0.001	%
MCV	32.1 ± 0.05	36.4 ± 0.2 *	0.1	fL
MCH	22.1 ± 1.9	19.1 ± 0.88	0.09	pg
MCHC	36.4 ± 0.2	31 ± 0.08	0.07	%
WBCs	18.11 ± 3.32	11.22 ± 1.4 *	0.001	×10^3^/µL
Neutrophils	8.99 ± 0.29	4.02 ± 0.08 *	0.004	×10^3^/µL
Lymphocyte	2.11 ± 0.55	4.1 ± 0.08 *	0.03	×10^3^/µL

RBCs, erythrocytes count; Hb, hemoglobin; PCV, packed cell volume; MCV, mean corpuscular volume; MCH, mean corpuscular hemoglobin; MCHC, mean corpuscular hemoglobin concentration; WBCs, total leukocytes count. Significant differences between the two groups are indicated by *: *p* < 0.05.

**Table 3:** Distribution and statistical analysis of single-nucleotide polymorphisms (SNPs) in immune (*IL-2*, *IL-6*, *TNFα*, *MCP-1*) and antioxidant-related genes (*SOD3*, *CAT*, *GPX*, *GST*) of apparently healthy (*n* = 180) and *Babesia*-infected (*n* = 63) calves in the New Valley Governorate, Egypt, including mutation type and amino acid changes.

Gene	SNPs	Healthy *n* = 180	Infected *n* = 63	Total *n* = 243	ChiSquare Value X2	*p*-Value	Type of Mutation	Amino Acid Number and Type
*IL-2*	C330T	-/180	34/63	34/243	112.9	0.001	Synonymous	I
*IL-6*	G224A	-/180	17/63	17/243	52.2	0.001	Non-synonymous	S to N
*TNFα*	G328A	-/180	28/63	28/343	90.4	0.001	Non-synonymous	V to M
	G488A	57/180	-/63	57/243	26	0.001	Non-synonymous	R to K
*MCP-1*	A93G	-/180	24/63	24/243	76	0.001	Synonymous	Q
	T180C	84/180	-/63	84/243	44.9	0.001	Synonymous	P
	A205G	67/180	-/63	67/243	32.3	0.001	Non-synonymous	I to V
	T270C	-/180	46/63	46/243	162.1	0.001	Synonymous	L
*SOD3*	A137G	93/180	-/63	93/243	52.7	0.001	Non-synonymous	N to S
*CAT*	T45C	129/180	-/63	129/243	96.2	0.001	Synonymous	Y
	T333C	107/180	-/63	107/243	66.9	0.001	Synonymous	F
*GPX*	T152C	-/180	34/63	34/243	112.9	0.001	Non-synonymous	L to P
*GST*	G147A	123/180	-/63	123/243	87.1	0.001	Synonymous	A
	C396G	-/180	25/63	25/243	79.6	0.001	Synonymous	P

Interleukin-2 (*IL-2*), Interleukin-6 (*IL-6*), Tumor Necrosis Factor-alpha (*TNF-α*), Monocyte Chemoattractant Protein-1 (*MCP-1*); Superoxide Dismutase 3 (*SOD3*), Catalase (*CAT*), Glutathione Peroxidase (*GPX*), and Glutathione S-Transferase (*GST*). A: Adenine, T: Thymine, C: Cytosine, G: Guanine.

**Table 4:** The observed genotype frequencies for each gene locus subjected to a chi-square (χ^2^) test to assess Hardy–Weinberg equilibrium and to characterize genotype distribution in healthy (*n* = 180) and *Babesia*-infected (*n* = 63) calves.

Gene	SNPs	Number	Genotyping Frequency			Allele Frequency		Chi-Square Calculated (X^2^)
			Healthy		Infected			
*IL-2*	C330T		CC	CT	TT	C	T	0.017
		Observed	209/0.86		34/0.14	0.63	0.37	
		Expected	96.47	113.24	33.29			
*IL-6*	G224A		GG	GA	AA	G	A	0.00000063
		Observed	226/0.93		17/0.07	0.74	0.26	
		Expected	133.65	92.34	17.01			
*TNFα*	G328A		GG	GA	AA	G	A	0.0003
		Observed	215/0.88		28/0.12	0.66	0.34	
		Expected	105.85	109.06	28.09			
	G448A		GG	GA	AA	G	A	0.1219
		Observed	57/0.23		186/0.77	0.12	0.88	
		Expected	3.40	51.27	188.08			
*MCP-1*	A93G		AA	AG	GG	A	G	0.2284
		Observed	219/0.90		24/0.10	0.70	0.30	
		Expected	119.07	102.06	21.87			
	T180C		TT	TC	CC	T	C	0.0034
		Observed	84/0.35		159/0.65	0.19	0.81	
		Expected	8.77	74.80	159.43			
	A205G		AA	AG	GG	A	G	0.0038
		Observed	67/0.28		176/0.72	0.15	0.85	
		Expected	5.47	61.97	175.57			
	T270C		TT	TC	CC	T	C	0.0288
		Observed	197/0.81		46/0.19	0.56	0.44	
		Expected	76.20	119.75	47.04			
*SOD3*	A137G		AA	AG	GG	A	G	0.0481
		Observed	93/0.38		150/0.62	0.21	0.79	
		Expected	10.72	80.63	151.66			
*CAT*	T45C		TT	TC	CC	T	C	0.0444
		Observed	129/0.53		114/0.47	0.32	0.68	
		Expected	24.88	105.75	112.36			
	T333C		TT	TC	CC	T	C	0.0079
		Observed	107/0.44		136/0.56	0.25	0.75	
		Expected	15.19	91.13	136.69			
*GPX*	T152C		TT	TC	CC	T	C	0.017
		Observed	209/0.86		34/0.14	0.63	0.37	
		Expected	96.47	113.24	33.29			
*GST*	G147A		GG	GA	AA	G	A	0.0142
		Observed	123/0.51		120/0.49	0.30	0.70	
		Expected	21.87	102.06	119.07			
	C396G		CC	CG	GG	C	G	0.00061
		Observed	218/0.90		25/0.10	0.68	0.32	
		Expected	112.36	105.75	24.88			

Interleukin-2 (*IL-2*), Interleukin-6 (*IL-6*), Tumor Necrosis Factor-alpha (*TNF-α*), Monocyte Chemoattractant Protein-1 (*MCP-1*); Superoxide Dismutase 3 (*SOD3*), Catalase (*CAT*), Glutathione Peroxidase (*GPX*), and Glutathione S-Transferase (*GST*). A: Adenine, T: Thymine, C: Cytosine, G: Guanine.

**Table 5:** Predicted group membership of calves based on diagnostic parameters: Classification of apparently healthy (*n* = 180) and diseased (*n* = 63) animals in the New Valley Governorate, Egypt.

		Predicted Group Membership		Total
		Healthy	Diseases	
Count	Healthy	180	0	180
	Diseased	0	63	63
%	Healthy	100	0.0	100.0
	Diseased	0.0	100	100.0

**Table 6:** Serum biochemical profiles of apparently healthy (*n* = 180) and *Babesia*-infected (*n* = 63) calves in the New Valley Governorate, Egypt.

Parameters	Healthy Calves	*Babesia* Infected Calves	*p* Values	Unit
Glucose	68.8 ± 0.8	48 ± 0.5 *	0.006	mg/dL
Cholesterol	89.6 ± 0.8	124 ± 1.5 *	0.001	mg/dL
Triglyceride	55.6 ± 0.8	82.6 ± 1.7 *	0.008	mg/dL
Total protein	6 ± 0.1	4.8 ± 0.1 *	0.001	g/dL
Albumen	4.4 ± 0.05	3.8 ± 0.08 *	0.001	g/dL
Globulin	1.3 ± 0.08	0.8 ± 0.05 *	0.001	g/dL
Urea	23 ± 0.5	31 ± 0.5 *	0.001	mg/dL
Creatinine	0.3 ± 0.02	0.7 ± 0.01 *	0.001	mg/dL
Total billirubin	0.2 ± 0.01	3.5 ± 1.5 *	0.001	mg/dL
Thyroxine	215 ± 7.6	165 ± 5.7 *	0.001	ng/mL
AST	54 ± 0.5	78 ± 0.5 *	0.001	U/L
ALT	30 ± 0.5	54 ± 0.5 *	0.001	U/L
GGT	6.7 ± 0.1	14.9 ± 0.5 *	0.001	U/L
LDH	102.3 ± 3.9	220.3 ± 15.7 *	0.001	U/L
cTnI	0.2 ± 0.04	1.3 ± 0.05 *	0.001	ng/mL
Calcium	9.6 ± 0.1	7.4 ± 0.2 *	0.001	mg/dL
Sodium	149.8 ± 1.2	131 ± 1.1 *	0.001	mmol/L
Potassium	5.3 ± 0.08	3.5 ± 0.05 *	0.001	mmol/L
Copper	1810 ± 7.9	14.1 ± 0.08 *	0.001	nmol/L
Zinc	79.9 ± 0.8	3.5 ± 0.05 *	0.001	nmol/L
Iron	165 ± 0.5	130 ± 0.5 *	0.001	nmol/L

AST, Aspartate aminotransferase; ALT, Alanine transaminase; GGT, Gamma-glutamyl transferase; LDH, Lactate Dehydrogenase; cTnI, Cardiac troponin I. Significant differences between the two groups are indicated by *: *p* < 0.05.

**Table 7:** Comparison of antioxidant and immune-related profiles between apparently healthy (*n* = 180) and *Babesia*-infected (*n* = 63) calves in the New Valley Governorate, Egypt.

Parameters	Healthy Calves	*Babesia* Infected Calves	*p* Values	Unit
GSH	28.3 ± 0.4	16.7 ± 0.7 *	0.001	mg/dL
CAT	42.6 ± 0.8	25 ± 0.5 *	0.001	U/mL
SOD	64.3 ± 1.4	25 ± 0.5 *	0.001	U/mL
TAC	102 ± 1.0	62.4 ± 0.6 *	0.001	mg/dL
MDA	0.81 ± 0.008	1.50 ± 0.008 *	0.001	mg/dL
NO	2.4 ± 0.1	6.4 ± 0.1 *	0.001	µmol/L
IL2	58 ± 1.1	167.6 ± 4.7 *	0.001	pg/mL
IL 6	38.2 ± 1.5	90 ± 38 *	0.001	pg/mL
IL 10	60 ± 1.1	119 ± 1.1 *	0.001	pg/mL
TNFα	49 ± 1	150.7 ± 2.6 *	0.001	pg/mL
IFNᵧ	30 ± 0.5	118 ± 2 *	0.001	pg/mL
MCP-1	40.2 ± 0.4	125 ± 0.5 *	0.001	pg/mL
SAA	3.7 ± 0.05	8 ± 0.08 *	0.001	mg/L
CRP	2.9 ± 0.3	9.6 ± 0.4 *	0.001	mg/L

GSH, glutathione reduced; CAT, Catalase; SOD, superoxide dismutase; TAC, total antioxidant capacity; MDA, malondialdehyde; NO, Nitric oxide; IL2, interleukin 2; IL6, interleukin 6; IL10, interleukin 10; TNFα, tumor necrosis factor alpha; INFγ, interferon gamma; MCP-1, Monocyte chemoattractant protein-1; CRP, C-reactive protein. Significant differences between the two groups are indicated by *: *p* < 0.05.

## Data Availability

The original contributions presented in this study are included in the article. Further inquiries can be directed to the corresponding authors.

## Supplementary Materials

The following supporting information can be downloaded at: https://www.mdpi.com/article/10.3390/vetsci13020176/s1, Figure S1: The original figure before formatting with data.
