# Supplementary material for: Integrated Molecular and Hematobiochemical Biomarkers for the Detection of Bovine Babesiosis in Holstein Calves
Source: Vet Sci. 2026 Feb 10;13(2):176. doi: 10.3390/vetsci13020176 (PMC12945156; doi:10.3390/vetsci13020176)
Supplement: Supplementary file 1 [file vetsci-13-00176-s001.zip › vetsci-4123882-supplementary.pdf]

Supplementary materials: Integrated Molecular and Hematobiochemical Biomarkers for the Detection of Bovine Babesiosis in Holstein Calves

Haifa Ali Alqhtani, Mohamed Marzok, Rasha yassin Elkhidr, Ahmed A. Elsayed, Safaa M. Barghash, Ahmed L. El-Naggar, Mohamed T. Ragab, Ahmed I. Ateya, Fatmah Ahmed Safhi and Wafaa A. Osman

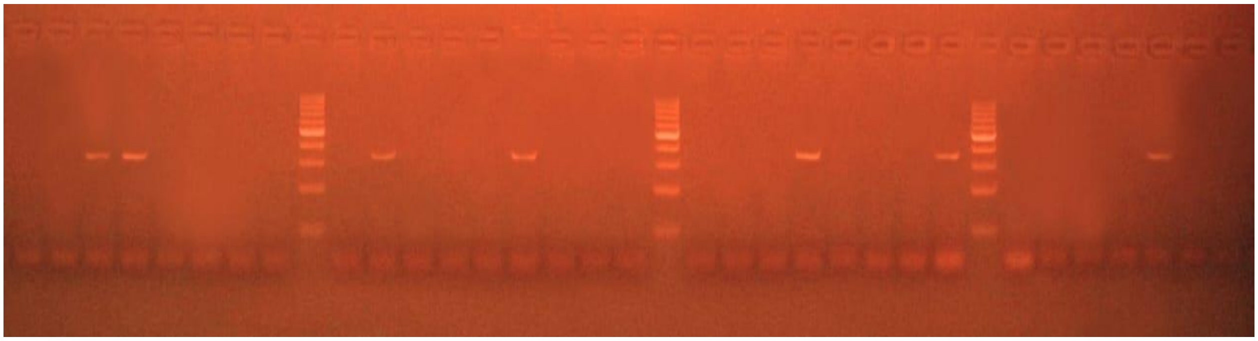

Figure S1. The original figure before formatting with data.
